# Complete genomes and comparative analyses of *Streptomyces* phages that influence secondary metabolism and sporulation

**DOI:** 10.1038/s41598-023-36938-z

**Published:** 2023-06-17

**Authors:** Sarah Kronheim, Ethan Solomon, Louis Ho, Michelle Glossop, Alan R. Davidson, Karen L. Maxwell

**Affiliations:** 1 Department of Biochemistry, University of Toronto, 661 University Avenue, Suite 1600, Toronto, ON M5G 1M1 Canada; 2 Department of Molecular Genetics, University of Toronto, 661 University Avenue, Suite 1600, Toronto, ON M5G 1M1 Canada

**Keywords:** Microbiology, Bacteriophages, Phage biology, Viral genetics, DNA sequencing

## Abstract

Bacteria in the genus *Streptomyces* are found ubiquitously in nature and are known for the number and diversity of specialized metabolites they produce, as well as their complex developmental lifecycle. Studies of the viruses that prey on *Streptomyces*, known as phages, have aided the development of tools for genetic manipulation of these bacteria, as well as contributing to a deeper understanding of *Streptomyces* and their behaviours in the environment. Here, we present the genomic and biological characterization of twelve *Streptomyces* phages. Genome analyses reveal that these phages are closely related genetically, while experimental approaches show that they have broad overlapping host ranges, infect early in the *Streptomyces* lifecycle, and induce secondary metabolite production and sporulation in some *Streptomyces* species. This work expands the group of characterized *Streptomyces* phages and improves our understanding of *Streptomyces* phage-host dynamics.

## Introduction

*Streptomyces* are Gram-positive bacteria that are ubiquitous in nature. These bacteria live primarily in soil but are also found in diverse habitats including oceans and deserts^1^. *Streptomyces* are well known for their prodigious production of secondary metabolites, with individual strains having the capacity to produce an estimated 20–40 different compounds^2–4^. These metabolites are not required for growth of the bacteria under laboratory conditions but are thought to provide fitness advantage in the natural environment. Many secondary metabolites inhibit the growth of competing species in the environment, including bacteria, fungi, and parasites^5,6^. Recent studies have also shown a role for secondary metabolites in defence against bacterial viruses known as bacteriophages (phages)^7–10^.

Phages are widespread in nature, occupying all environments where bacteria are found. They are thought to outnumber their bacterial hosts by a factor of ten and their predation of bacteria plays important roles in shaping microbial communities^11,12^. *Streptomyces* phages were isolated as far back as the 1970s, and phages such as ϕC31, ϕBT1, and SV1 have been used to develop important genetic tools that can be used to manipulate *Streptomyces*^13,14^. Both phage infection and secondary metabolite production are linked to the *Streptomyces* lifecycle, and certain secondary metabolites have been shown to inhibit phage replication^7–10,15–17^. However, the relationship between *Streptomyces* secondary metabolism and phage infection has not been well characterized.

In this work we report the characterization and genomic analysis of twelve *Streptomyces* phages. Comparative analyses show that these phages, isolated from diverse geographic locations, share closely related genome sequences and cluster with the previously characterized *Streptomyces* phages R4 and ϕJoe. These phages display broad host ranges and can efficiently replicate in many *Streptomyces* species. Whole genome analyses of these phages show a variable accessory genome that likely contributes to host range and the differing abilities of these phages to induce secondary metabolite production and early sporulation.

## Results

### *Streptomyces* phage sequencing and genome comparisons

Previous investigations in our lab characterizing chemical anti-phage defence^7^ used a collection of *Streptomyces coelicolor* phages (ϕScoe phages) isolated from geographically diverse soil samples. Negatively stained electron microscopy images showed that these phages belong to the order *Caudovirales*, with double-strand DNA genomes contained inside icosahedral heads and long, non-contractile tails. To gain insight into this phage collection, we determined their genome sequences using Illumina NextSeq 2000 short-read technology. Each genome was assembled into a single contig, with > 86% of the reads mapped to each sequence. The genomes were annotated using automated gene predictions followed by careful manual inspection using our in-house phage database for functional annotations.

**Figure 1:**
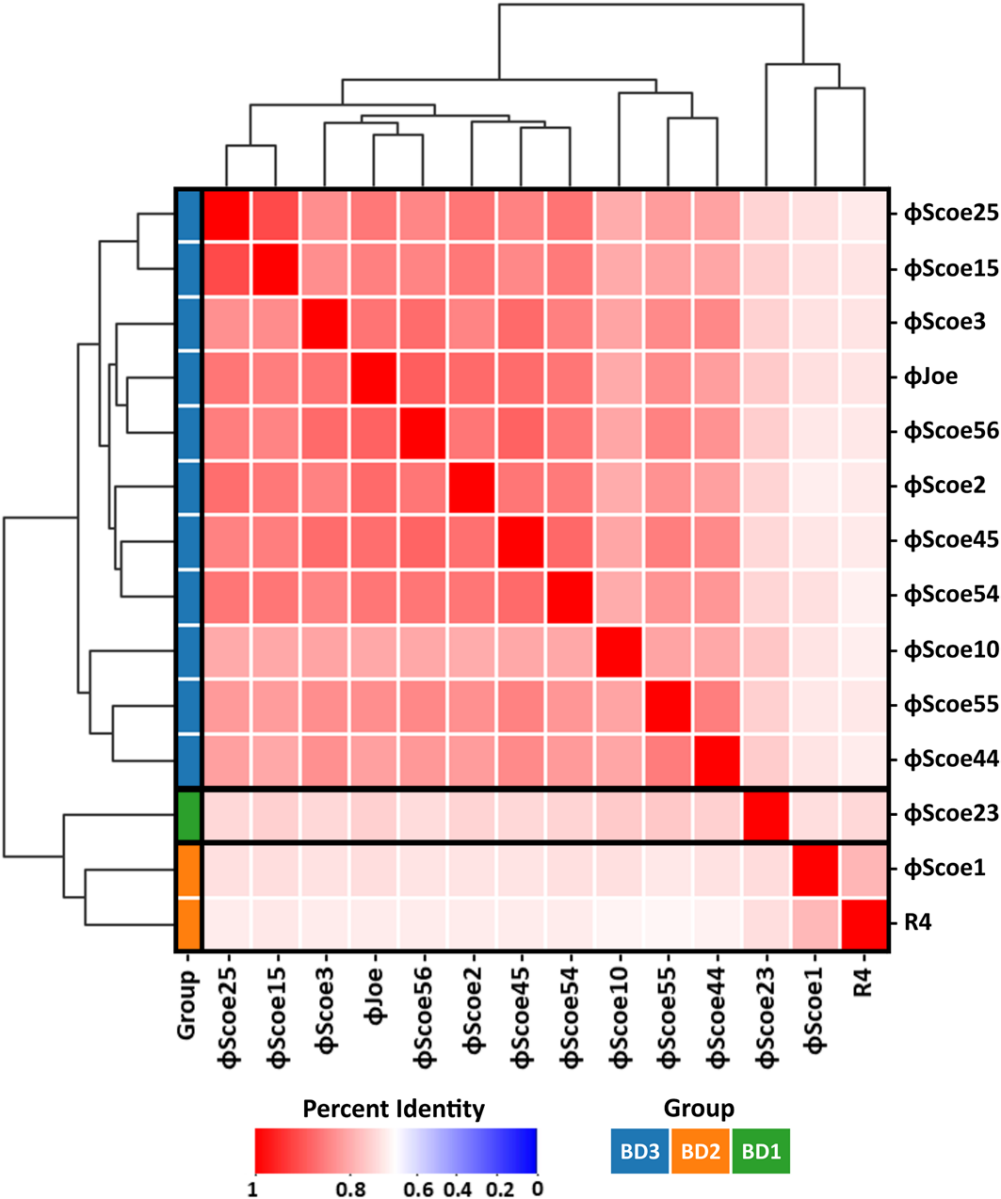
Heatmap of the average nucleotide identity (ANI) values for the twelve newly sequenced phage genomes with previously reported *Streptomyces* phages ϕJoe and R4. The shading represents the percent sequence identity shared by the noted phages. The Actinophage Database BD subgroup each phage belongs to is noted, and phages in the same subgroup are boxed.

**Figure 2:**
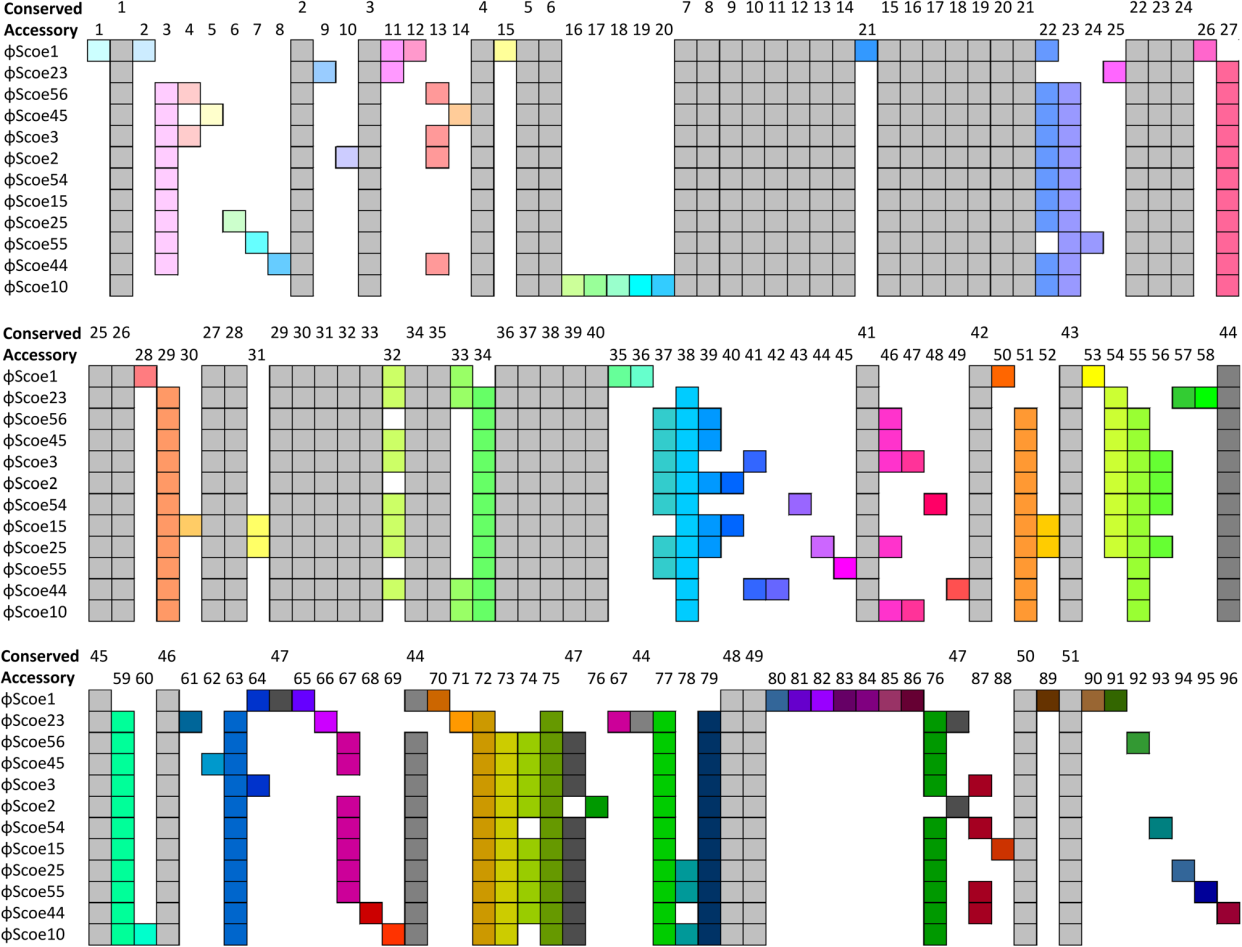
Conserved and accessory gene map. Alignment of the phage genomes shows that these phages are closely related. Each box represents a single open reading frame; conserved genes shown in grey, and the non-conserved “accessory genes” are shown in colour. Different vertical positions and colours for the accessory genes indicate that they encode distinct protein families. Darker grey boxes indicate genes that are conserved but appear in different places in these genomes. Genes are grouped if they share > 30% identity or share a specific confirmed biological function (e.g., receptor binding proteins are grouped by sequence identity while “serine integrase” is a group despite high sequence diversity). Genomes are organized by average nucleotide identity. See Supplementary Table S1 for details about specific gene annotations.

DNA-DNA comparisons performed using Pyani^18^ revealed that the twelve ϕScoe phage genomes shared pairwise nucleotide identities ranging from 73 to 91% across the complete genome sequences (Fig. 1, Supplementary Fig. S1a). The ϕScoe phage genome lengths varied in size from 48 to 51 kb, and each was found to contain between 75 and 84 predicted open reading frames (ORFs) (Table 1). Of these ORFs, 51 were found to be conserved among all twelve phages (Fig. 2, Supplementary Fig. S1b, Supplementary Table S1). The genome organization of these phages is similar to previously characterized *Streptomyces* phages, encoding the functional modules necessary for Siphophage particle formation (DNA packaging, capsid and tail assembly), cell lysis, DNA replication, as well as an immunity repressor and serine integrase that suggest a temperate lifestyle for these phages (Supplementary Table S2). Genome comparisons with previously characterized phages revealed striking similarity to *Streptomyces* phages R4^19^ and ϕJoe^20^, with average pairwise nucleotide identities of 71–78% and 73–89% respectively (Fig. 1, Supplementary Fig. S1). The R4-like group of phages appears to be the most abundant group of *Streptomyces* phages^21^, falling within the BD family at the Actinobacteriophage Database^22^.

**Table 1:** Phage genome features.

Phage	Genome size (kb)	GC content (%)	Predicted ORFs	Lifestyle	Genbank accession
ϕScoe1	51	66.83	80	Temperate	OQ672741
ϕScoe2	49.4	65.77	79	Temperate	OQ672744
ϕScoe3	48.3	65.6	81	Temperate	OQ672747
ϕScoe10	51.1	65.23	80	Temperate	OQ672742
ϕScoe15	49.4	66.07	80	Temperate	OQ672743
ϕScoe23	49.8	65.84	75	Temperate	OQ672745
ϕScoe25	49.4	66.13	84	Temperate	OQ672746
ϕScoe44	48.8	65.08	80	Temperate	OQ672748
ϕScoe45	48.8	65.57	80	Temperate	OQ672749
ϕScoe54	48.7	65.83	78	Temperate	OQ672750
ϕScoe55	48.5	65.17	77	Temperate	OQ672751
ϕScoe56	48.4	65.47	78	Temperate	OQ672752

### Conserved *Streptomyces* phage proteins

Both R4 and ϕJoe are temperate phages, which means that they can pursue either a lytic or lysogenic lifestyle following phage infection. Temperate phages require an immunity repressor to control the balance between lysis and lysogeny. They do so by downregulating promoters required for the lytic pathway. Each of the ϕScoe phages was found to encode a repressor protein between the head–tail–lysis genes and the putative early region genes. These repressor proteins share sequence identities of 64–90% with the repressor proteins found in *Streptomyces* phages R4 and ϕJoe (Supplementary Fig. S2). Phage R4 has an atypical regulatory mechanism that silences transcription from the prophage, known as the repressor-stoperator regulatory system^21^. This type of repressor regulates transcription initiation at an early lytic promoter, but also acts at many sites across the phage genome, blocking the movement of RNA polymerase and thereby helping to maintain lysogeny. Bioinformatic analyses revealed eight or more R4 stoperator binding sites in intergenic regions of the ϕScoe phages (Supplementary Table S3), suggesting that this mechanism of repression is conserved across this family of phages.

Most temperate phages encode an integrase protein whose activity mediates integration and excision of the prophage during the lysis-lysogeny decision. The integrases of many *Streptomyces* phages have been shown to belong to the large serine recombinase family^23^. In keeping with this, the ϕScoe phages characterized in this work each encode a serine integrase. These proteins are highly variable in sequence across the twelve phages, with pairwise sequence identities ranging from 13 to 91% (Supplementary Fig. S3a). Two groups of phages have more closely related integrases: phages ϕScoe2, ϕScoe15, and ϕScoe55 share 75–91% identity with each other and the previously characterized ϕJoe integrase, while ϕSoce25, ϕScoe56, ϕScoe23, ϕSoce45, and ϕScoe10 share 64–83% identity among their integrases. During phage integration, the serine integrase protein acts at specific sites: the *attP* site in the phage and the *attB* site in the bacterial chromosome. Phages that encode similar integrases and *attP* sites use the same bacterial *attB* sites for integration^24^. To identify potential *attP* sites in the ϕScoe phages we queried their sequences with known *attP* sequences from previously characterized actinophages R4^25^, ϕJoe^20^, ϕC31^26^, ϕBT1^27^, TG1^28^, and SV1^14^ (Supplementary Fig. S3b). Sequences related to the ϕJoe *attP* site were identified upstream of the integrase genes in phages ϕScoe2, ϕScoe15, and ϕScoe55 (Supplementary Fig. S3c). These four phages encode integrases that share high sequence identity and are more distantly related to the other serine integrases found in the ϕScoe group of phages. The *attP* sites were not identified for the remaining phages.

**Figure 3:**
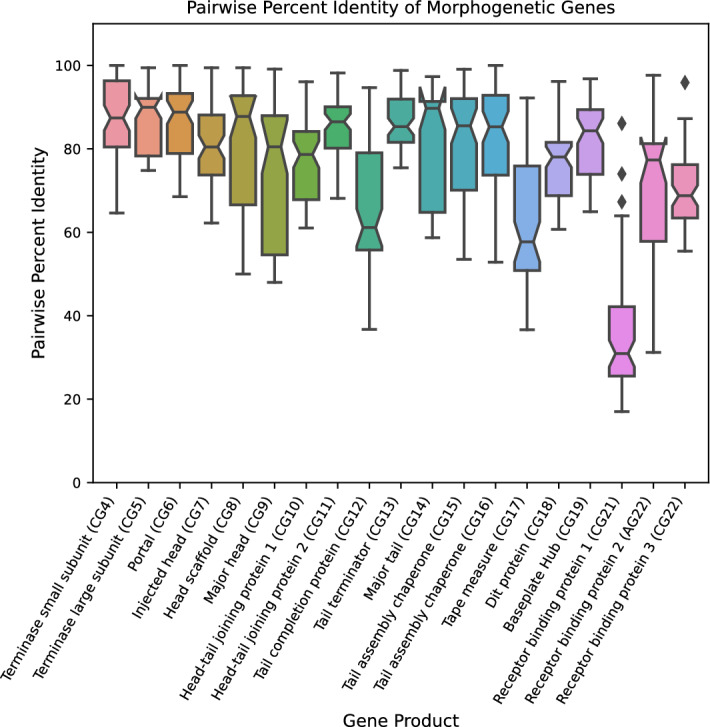
Morphogenetic gene conservation. Box-and-whisker plot of pairwise percent sequence identities for each annotated morphogenetic gene that is conserved among at least ten phages. Receptor binding protein 2 is conserved in all phages except ϕScoe23 and ϕScoe55. The box bounds the IQR divided by the median, and the whiskers extend to 1.5 × IQR beyond the box. Outliers are shown as individual points.

The genes that are required for the assembly of the head and long, non-contractile tail of the siphophage virion are typically found in a cluster with a conserved gene order. As previously described^29^, we combined analysis by HHpred^30^ and gene position to annotate genes encoding morphogenetic functions, such as the large and small terminases, the capsid and portal proteins, head–tail joining proteins, and tail tube protein, that are conserved among most long-tailed phages (Supplementary Table S2). These functions were named according to Casjens et al.^29^. Proteins comprising the distal end of the tail, referred to as the baseplate or tail tip complex, vary among different types of long-tailed phages. In this region, the ϕScoe phages resemble other siphophages that infect Gram-positive species, such as *Lactococcus* phage TP901-1. Accordingly, the first conserved gene in the baseplate region encodes the Distal Tip (Dit) protein and the second gene encodes a protein that structurally resembles the baseplate hub protein (BH2) of myophages^31^ and is often referred to as Tal in the Gram-positive siphophage literature^32^. Proteins encoded by genes CG21, CG22, and AG22 are predicted by HHpred to resemble receptor binding proteins found in other siphophages; thus, these proteins have been assigned that function (Supplementary Fig. S1). These receptor binding proteins share average pairwise sequence identities of 34–70%. The greatest variability observed in the morphogenetic region was noted in the proteins that comprise the tail tip (Fig. 3). The variability in the tail tip proteins may contribute to the bacterial host range differences noted for these phages.

### Non-conserved phage proteins

In addition to the highly conserved genes required for replication and assembly of the phage particle, the ϕScoe phages encode many genes that are not conserved among all members of this group, which we refer to as accessory genes (AGs). Between the twelve phages there are a total of 96 different accessory genes that are present in at least one phage. Eleven accessory genes are present in every phage except ϕScoe1. Nine of these genes are of unknown function, and the remaining two encode for Lsr2 and Ocr (Overcome classical restriction) proteins (Fig. 2, Supplementary Table S1). Lsr2 is a nucleoid-associated protein that binds AT-rich regions of DNA; it plays a role in silencing prophages in *Streptomyces venezuelae*^33^ and *Corynebacterium glutamicum*^34^. As the ϕScoe phages encode repressor proteins that maintain lysogeny, the Lsr2 proteins they encode may play a role in microbial warfare by inhibiting the host encoded Lsr2 proteins. Lsr2 also influences the dynamics of the replication machinery and the duration of DNA synthesis in mycobacteria^35^, suggesting a possible role for the phage Lsr2 proteins in disrupting host processes to subvert them in a way that benefits the phage. Ocr is a protein found in phage T7 that counters restriction-modification and BREX anti-phage defences^36,37^ and likely plays a similar role in these phages, perhaps to counter the BREX phage growth limitation (Pgl) system^38^ found in *S. coelicolor*.

Fifty-nine genes are found in only one phage (Fig. 2, Supplementary Table S1). Of these genes, only two have predicted functions; ϕScoe23 encodes a DNA polymerase III (AG58), and ϕScoe45 contains an endonuclease (AG62) in addition to the HNH-endonuclease that all twelve phages share. Phage ϕScoe10 contains a cluster of five accessory genes in the morphogenetic region, between the portal protein and capsid maturation protease (Fig. 2). Four of these genes are annotated as hypothetical proteins, while AG17 shares 62% sequence identity with a tail fibre protein from an *Agrobacterium* phage, suggesting a potential role in bacterial host range. Most accessory genes in this group of phages are located downstream of the morphogenetic region. These genes share synteny, but there are several local rearrangements in this region (Fig. 2). For example, AG67 in ϕScoe23 and AG76 in ϕScoe2 are present in different relative locations than in the remaining phages that encode these proteins. This pattern is observed for conserved genes as well. For example, conserved gene CG47 appears at different locations in phages ϕScoe1, ϕScoe2, and ϕScoe23 as compared to the rest of the phages.

### Phage infection induces secondary metabolite production and early sporulation

**Figure 4:**
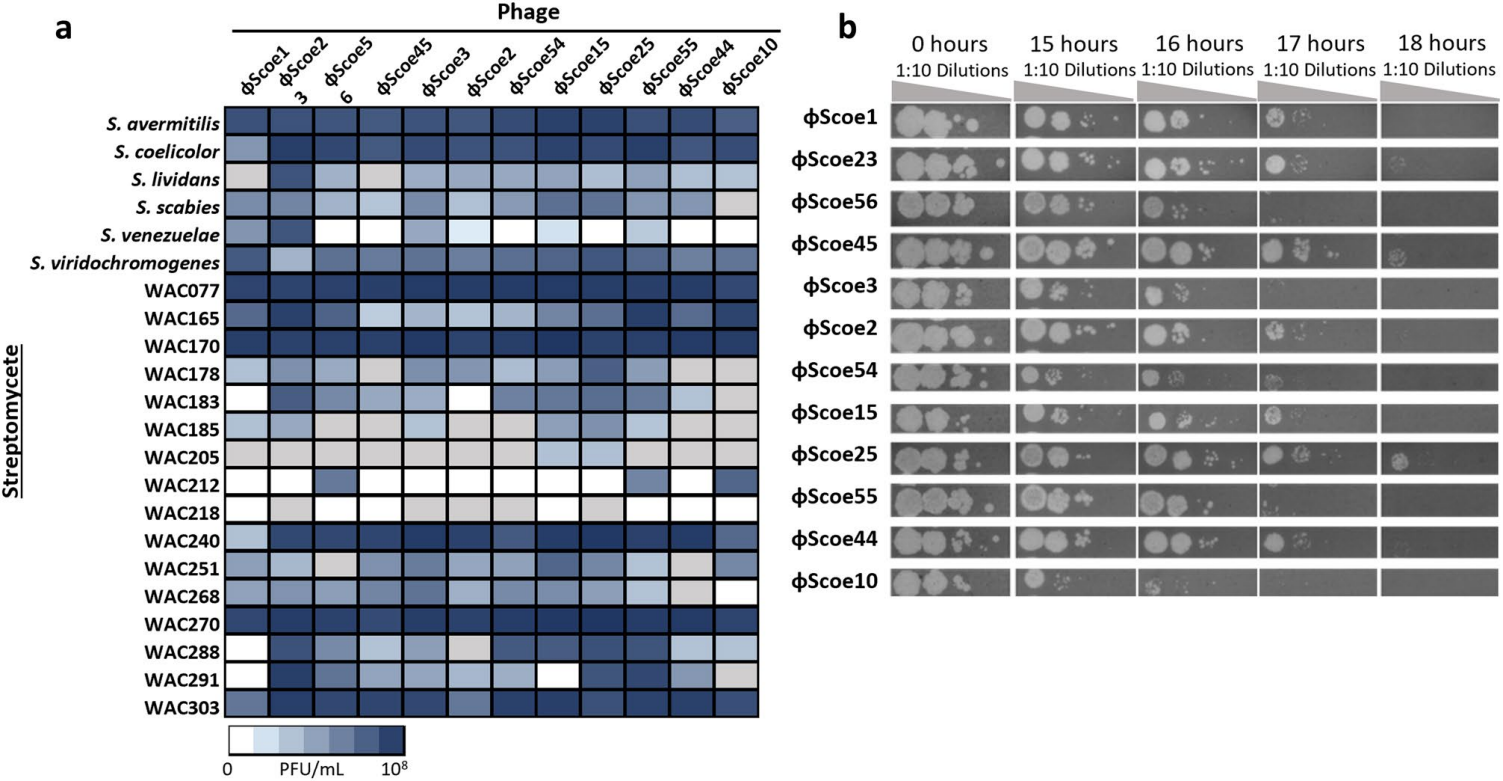
Infection profiles for the ϕScoe phages. (**a**) Host range of the ϕScoe phages was assessed using six well characterized *Streptomyces* species and 16 strains from the Wright Actinomycete Collection (WAC). Boxes are colored based on the apparent phage titer on each strain, from 0 to 10^8^ PFU/mL. Grey boxes indicate clearance of the bacterial lawn without visible plaques. (**b**) Infection of *S. coelicolor* over time. Ten-fold serial dilutions of each phage (starting titer of 3 × 10^4^ PFU/mL) were plated on a lawn of *S. coelicolor* spores after 0, 15, 16, 17, or 18 h of growth at 30 °C.

**Figure 5:**
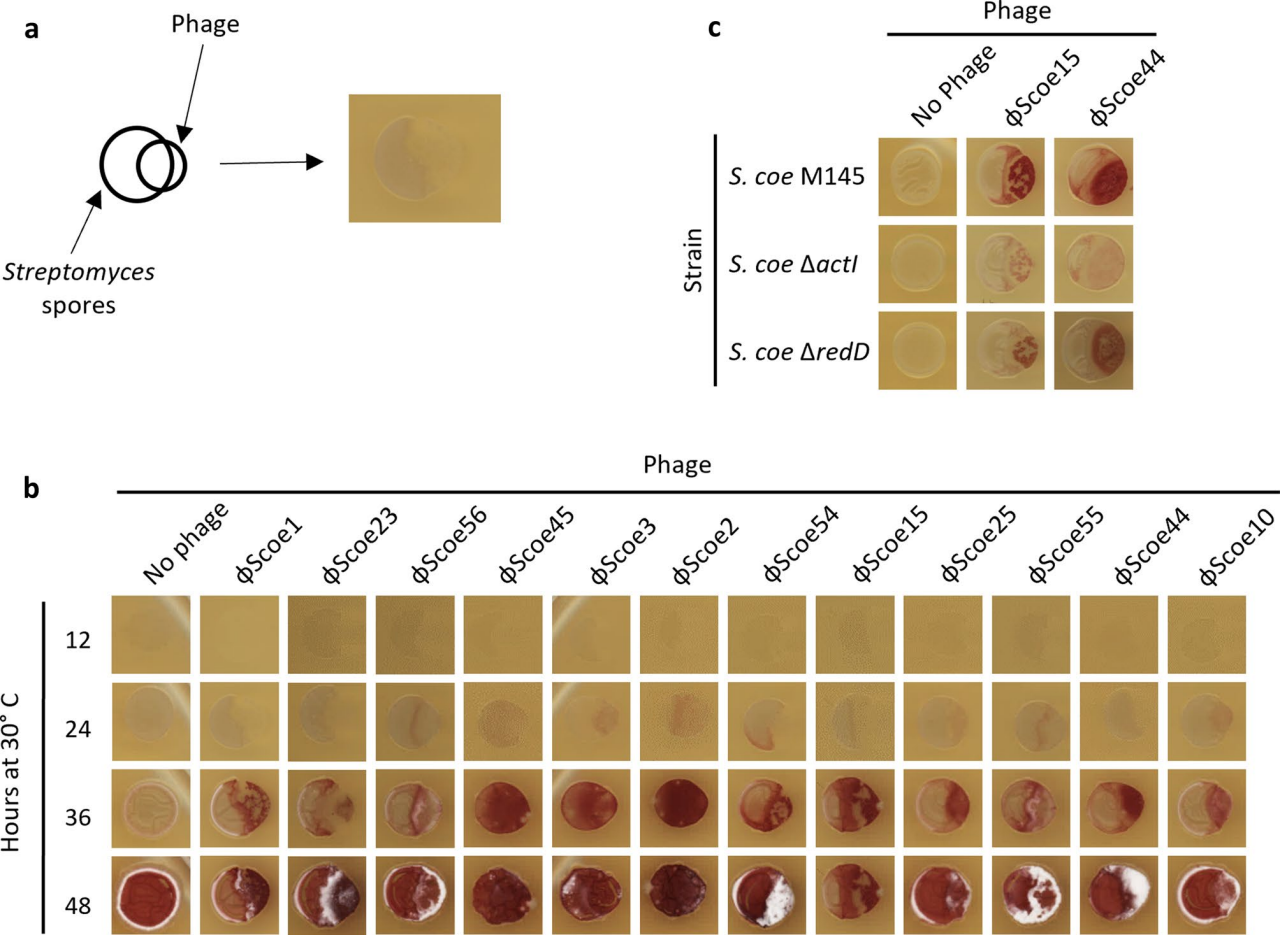
Phenotypic changes in *S. coelicolor* following phage infection. (**a**) *S. coelicolor* spore stocks were applied to agar plates and phage lysates were deposited so that they partially overlapped the spores to allow for infection to proceed from one side of the bacterial colony. (**b**) Images of ~ 10^4^ spores of *S. coelicolor* M145 spotted alone and after infection of one side of the bacterial colony by each phage at 12, 24, 36, and 48 h of growth. (**c**) Images of *S. coelicolor* M145, Δ*actI*, and Δ*redD* after 36 h of growth, alone or when challenged by phages ϕScoe15 and ϕScoe44. Images were captured using an Epson Canada Perfection V850 Pro Scanner.

**Figure 6:**
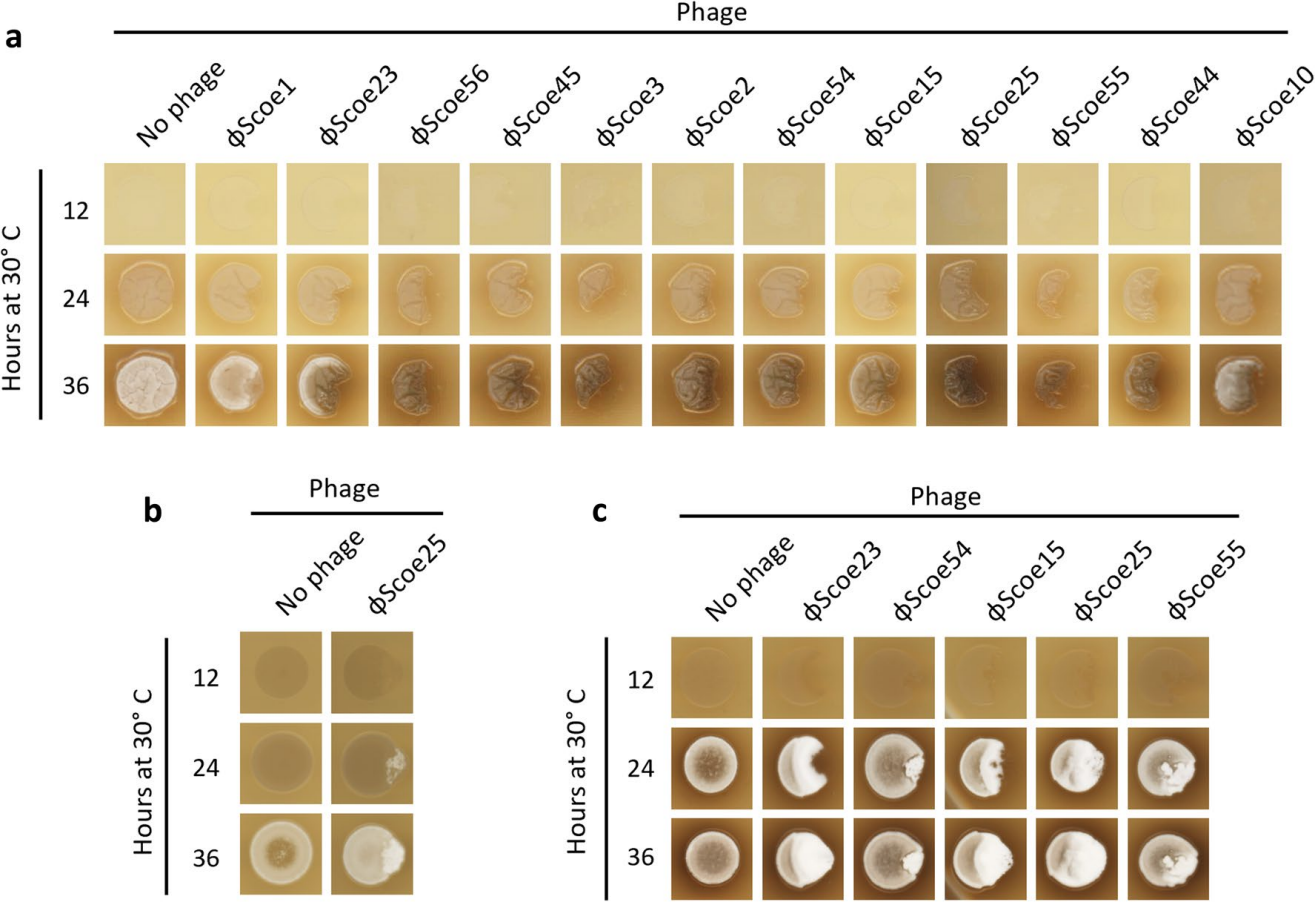
Infection phenotypes of other *Streptomycetes.* (**a**) Images of WAC170 colonies following challenge by the ϕScoe phages were recorded at 12, 24, and 36 h of growth. (**b**) Images of WAC178 following infection by phage ϕScoe25 taken after 12, 24, and 36 h of growth. (**c**) Images of WAC288 spotted alone and following infection by phages ϕScoe23, ϕScoe54, ϕScoe15, ϕScoe25, and ϕScoe55 taken after 12, 24, and 36 h of growth. All images were captured using an Epson Canada Perfection V850 Pro Scanner.

While the ϕScoe group of phages share many genes across the full length of the genome, the variable accessory gene complements suggested that they may have different host ranges. To address this question, the twelve phages were propagated to high titer in *S. coelicolor* and their plating efficiencies were assessed on a collection of 21 *Streptomyces* species. Each phage showed a unique infection profile (Fig. 4a), with all phages able to efficiently replicate in more than half of the species tested. In some cases, we observed clearings that did not resolve into plaques (grey shading). This suggests that the phages infect but are unable to proceed through the replication cycle, perhaps because the strain lacks a host factor that the phage needs to replicate, or because it encodes a defence system that targets the phage intracellularly. As we previously determined^7^, some species are highly resistant to phage infection, while others are very sensitive. No patterns between phage gene conservation and host range could be discerned. However, we noted that some *Streptomyces* species appear to upregulate secondary metabolite production in the presence of phage challenge as shown by the production of coloured compounds (Figs. 5, 6).

Secondary metabolite production^39^ and protein-based anti-phage defences^40–42^ are upregulated at high cell density. *Streptomyces* have a complex developmental lifecycle, in which they grow from spores into a branching network of young mycelia. The young mycelia grow into mature mycelia over time, moving into exponential growth approximately 12–14 h post-inoculation^43,44^. The cells move into a transitionary phase after approximately 18 h of growth and undergo a developmental switch, after which they begin producing specialized metabolites and aerial hyphae which mature into spores^45,46^. To determine how this developmental process affected the ϕScoe phages, we determined how efficiently they could initiate infection and replicate in *S. coelicolor* over time. The phage lysates were diluted to approximately 3 × 10^6^ plaque forming units (PFU) per mL and plated on cells that had been grown for various amounts of time following spore germination. Most of the phages maintained normal plating efficiency until ~ 16 h, with some (e.g. ϕScoe2 and ϕScoe45) still able to plate on cells that had been grown for 18 h before the phages were applied (Fig. 4b). By contrast, ϕScoe56 formed plaques poorly at 15 h, suggesting that this phage was more sensitive to some cellular factor that is differentially expressed during the *Streptomyces* lifecycle. None of the phages were able to efficiently replicate in mature mycelia, which is consistent with previous work showing that *Streptomyces* phages replicate best in exponentially growing cells^17,45,46^.

We also investigated the effects of ϕScoe phage infection on bacterial growth. In this assay ~ 100 phages were applied to a DNB-agar plate adjacent to a spot containing ~ 1000 *S. coelicolor* spores, so that the two samples were partially overlapping (Fig. 5a). These cultures were incubated at 30 °C and bacterial growth was monitored using a high-resolution scanner, with images captured every 12 h for a 120 h incubation period. Bacterial growth was initially inhibited by phage infection where the bacterial spores and phages overlapped (Fig. 5b). At 24–36 h bacterial growth began to appear where the initial phage infection had taken place, radiating out from the larger uninfected colony into the zones of clearing. The cells that grew in these areas, which had initially been completely lysed by the activity of the phages, appeared to be resistant to phage infection—this could be either through mutations in the bacteria that provide resistance, through the formation of lysogens, or perhaps through a developmentally mediated resistance mechanism. To determine if bacterial mutations or lysogen formation was the reason for the observed resistance, we isolated colonies from these resistant cells and prepared spore stocks to allow us to test for resistance to further phage infection. Following germination of the spores, no phage resistance was observed and PCR-based screening of the previously phage resistant cells showed that they did not contain prophages. These data suggest that resistance that arises following phage challenge is through some developmentally regulated factor.

The infection and replication of the ϕScoe phages in *S. coelicolor* colonies triggered the production of a red-coloured secondary metabolite in response to the phage challenge (Fig. 5b). *S. coelicolor* is known to make two red metabolites, actinorhodin^47^ (red or blue depending on pH) and undecylprodigiosin^48^. To determine which of these metabolites was being induced by phage infection, we monitored growth of *S. coelicolor* strains that harboured mutations in *actI*^49^ and *redD*^50^, which do not produce actinorhodin or undecylprodigiosin, respectively. Infection of wild type *S. coelicolor* by all ϕScoe phages induced high levels of red metabolite at 36 h (Fig. 5b, c). This phenotype was greatly abrogated in the *actI* mutant, suggesting that actinorhodin is the major contributor to this phenotype (Fig. 5c). This result is consistent with recent work that showed several other *Streptomyces* phages induced actinorhodin production upon phage infection^51^. Infection of *S. coelicolor* by several of the ϕScoe phages also appeared to trigger early sporulation in infected colonies as shown by the appearance of a fuzzy white colony morphology at later timepoints (Fig. 5b).

To determine if these metabolite and sporulation effects are a general response to phage infection, we challenged 15 other *Streptomyces* species with phages that were able to plate to high titer and looked for visible cellular responses. Phage infection induced production of a dark brown metabolite in WAC170 (Fig. 6a). Notably, induction of this metabolite appears to be phage-specific; although ϕScoe1 visibly infected and killed a portion of the bacterial colony it did not induce production of this secondary metabolite. Strains WAC178 and WAC288 both showed signs of early sporulation in response to phage infection (Fig. 6b,c). As *Streptomyces* phages replicate only in exponentially growing cells^17,45,46^, this hastening of sporulation may be an adaptive response to allow cells within the colony to become phage resistant and limit the phage epidemic.

## Discussion

*S. coelicolor* has long been used as the model organism for understanding *Streptomyces* biology. Studies of its lifecycle provided critical insight into morphological development pathways and the production of secondary metabolites. In this work we present the genome sequences of twelve *S. coelicolor* phages and show that their activities affect both sporulation and secondary metabolite production. Despite the fact that these phages were isolated from geographically diverse sources spanning multiple continents^7^, they showed remarkable sequence conservation across their complete genomes, as well as to previously characterized phages^19–21^. This is likely a result of the selective pressure imposed by the bacterial strain on which these phages were initially isolated. The remarkable similarity of these genomes highlights the common worldwide gene pool that phages draw from.

Sequence analyses revealed that this group of phages fall within a single cluster and share high genetic similarity with a variety of other phages infecting *Streptomyces*. The variability of plating behaviours, particularly with respect to secondary metabolite production and the early sporulation effects, is surprising in light of this high degree of sequence conservation. These observed effects on the bacterial host following infection are likely the result of the highly variable accessory gene complement carried by these phages; some of the accessory genes are shared among many of the phages, while others are unique to one. The variable accessory gene content of these phages makes it difficult to predict which ones are contributing to these effects.

While only visible phenotypic changes in bacterial colony morphologies—production of coloured secondary metabolites and early sporulation—following phage challenge were monitored in this study, there are likely many other changes induced by this group of phages. Many Streptomyces species, including *S. coelicolor*, produce a wide variety of uncolored metabolites; any changes in production of these compounds in response to phage infection would not have been detected in our assays. However, we believe that induction of secondary metabolites upon phage challenge is likely a frequently occurring response to phage infection in *Streptomyces*. Phage infection may act as a global inducer of specialized metabolism in *Streptomyces*, or it may induce specific metabolites. The reasons for the increased production of secondary metabolites are not yet known, but they may act as a defence against other incoming phages or serve as a warning to surrounding cells of phage infection. More detailed studies will be required to determine what metabolites are induced and the function of these metabolites in the context of phage infection. As secondary metabolite production is tightly linked to sporulation in *Streptomyces*, these effects may also be linked to the acceleration of sporulation observed in *S. coelicolor,* WAC178, and WAC288 in response to phage infection. Our results, in combination with recent work showing induction of chloramphenicol and accelerated sporulation in *S. venezuelae* in response to phage infection^17^, suggest a variety of developmental changes are triggered in *Streptomyces* in response to phage infection. Understanding the complex interplay between *Streptomyces* and the phages that infect them has the potential to deepen our understanding of *Streptomyces* bacteria, uncover novel secondary metabolites, and improve our understanding of the role of secondary metabolites in the phage-host evolutionary arms race.

## Materials and methods

### Phage isolation and maintenance

Phages were isolated from soil samples using overnight enrichment with *Streptomyces coelicolor* M145 (ϕScoe45 and ϕSoce54), WAC212 (ϕScoe10), or *Streptomyces avermitilis* SUKA22 (remaining phages) as reported previously^7^. Stocks were maintained by propagation on *S. avermitilis* (ϕScoe1) and *S. coelicolor* (all remaining phages) in Difco nutrient broth supplemented with 4 mM CaCl_2_ and 0.5% glucose (DNB medium). Phage lysates were stored at 4 °C.

### DNA isolation

For each phage, 100 mL of DNB medium was inoculated with 10^9^ spores of *S. coelicolor* and 10^7^ plaque forming units (PFU) of the relevant phage. Cultures were grown at 30 °C for 16 h after which time the culture supernatant was collected. Phages were precipitated via overnight incubation at 4 °C with 20% PEG 8000 and precipitate was collected by centrifugation at 11,000×*g* for 10 min at 4 °C. Phages were resuspended in 10 mM Tris pH 7.5, 10 mM MgSO_4_, 70 mM NaCl, and 1 mM CaCl_2_, and DNA was extracted using phenol–chloroform as previously described in Bondy-Denomy et al.^52^.

### Genome sequencing and assembly

Phage genomes were sent to the Microbial Genome Sequencing Center in Pittsburgh for sequencing via NextSeq2000. Paired end fastQ files with reads of 30–150 bp long were obtained, and 50,000 reads from each phage was subsampled and assembled using Newbler (GS De novo assembler). Remaining reads were added to the assembly and the resulting single contigs for each phage were then manually curated using Consed^53^. Bowtie2 was used to determine the percent of reads that aligned to each assembly^54^.

### Genome annotation and comparison

Open reading frames (ORFs) were identified and annotated using DNA Master^55^, Glimmer^56^, and GeneMark^57^ software, BLAST searches^58^ and visual inspection. The Phage Annotation Toolkit, our in-house annotation database was used to further annotate morphogenetic phage genes. The annotated genomes were deposited in GenBank (Table 1).

Alignment of the genomes was performed using Clinker^59^ to determine shared percent identity of each ORF between these phages with a cut-off of 30% identity. Conserved genes are present in every phage and are defined as genes that either share > 30% identity or are annotated with the same specific function through BLAST search (i.e. serine integrase). Phage lifecycle was determined by visual inspection and identification of serine integrases in all phages and attachment sites of several phages using BLAST.

Phage genomes were assessed for the presence of known stoperator sites via MEME suite^60^. The hits were then analyzed for location and summary table was generated to show intergenic hits of each known stoperator sequence in each ϕScoe phage. Several regions where characterized stoperators are located were aligned to equivalent regions in each ϕScoe phage and a percent identity matrix was generated via Clustal Omega^61^.

Pairwise average nucleotide identities (ANI) were calculated with the twelve ϕScoe phages and characterized *Streptomyces* phages R4 and ϕJoe using pyani 0.2.9^18^ with ANIb method. The output average nucleotide identity matrix was used for clustering and display via Seaborn clustermaps^62,63^.

To find phage attachment sites, intergenic DNA flanking the integrase of each phage was BLASTed against published attP sites from 6 characterized *Streptomyces* phages. Significant hits were examined manually and matched up to the characterized attP site, and sequences with > 70% identity over the entire attP site were reported.

### Host range and efficiency of plating

Phages were propagated on *S. avermitilis* (ϕScoe1) or *S. coelicolor* (remaining phages). Ten-fold serial dilutions were plated on DNB plates overlaid with 0.5% (w/v) top agar containing approximately 10^6^ spores of one of six known Streptomycetes or 16 strains from the Wright Actinomycete Collection. The titre of each phage on each strain was recorded, as was the appearance of clearings that did not resolve into individual plaques. Experiments were performed with three biological replicates.

### Infection time-course

All phages were propagated on *S. coelicolor*, then diluted to 3 × 10^6^ PFU/mL in liquid DNB. Plates and serial dilutions were prepared as described for host range with DNB top agar containing 3 × 10^7^ *S. coelicolor* spores. Dilutions were spotted on fresh overlays as time 0, following which the plates were place at 30 °C. Fresh phage dilutions were prepared the following day and spotted at 15, 16, 17, and 18 h post overlay with incubation at 30 °C between each timepoint. The resulting plaques were counted to determine phage titre at each hour and the experiment was performed in triplicate.

### Phage infection phenotypes

To investigate phenotypic changes in *Streptomyces* upon phage infection, preliminary screening was performed using a plate pinner. Spores of 16 *Streptomyces* species were diluted to ~ 10^6^ spore forming units per mL (sfu/mL) and aliquoted into wells of a 96 well plate. Phages were diluted to ~ 10^7^ plaque forming units per mL (PFU/mL) and tenfold serial dilutions of each were aliquoted into descending wells of a 96 well plate, with no phage added to the bottom well of each column. ISP-2 agar supplemented with 4 mM CaCl_2_ and made with either all MiliQ water or half MiliQ and half tap water (ISP-2 and ISP-2 tap), and MYM agar supplemented with 4 mM CaCl_2_ and made with MiliQ water only (MYM MiliQ) were poured into Singer PlusPlates and allowed to dry. Approximately 0.5 mL each of spores were spotted onto a plate containing each medium at 384 spot density using a Singer ROTOR HDA pinning robot and Singer 96 density RePads and allowed to dry. Approximately 0.5 mL of each phage dilution was then spotted on top of each spore spot using the Singer ROTOR HDA and RePads. Plates were allowed to dry, and then placed facedown onto the glass of an Epson Canada Perfection V850 Pro Scanner—B11B224201. Pictures were taken using the scanner at 6 h increments for 120 h, controlled by Raspberry Pi and code adapted from that described in French et al.^64^. Species used include *S. coelicolor, S. avermitilis, S venezuelae*, WAC077, WAC170, WAC178, WAC185, WAC205, WAC212, WAC218, WAC240, WAC251, WAC268, WAC270, WAC288, WAC291, and WAC303. The experiment was performed with four technical replicates per pinned plate and observed phenotypic changes were confirmed as below.

To confirm the phenotypic changes observed using the scanner, ~ 1000 spores of *Streptomyces* strains of interest were plated on the medium on which phenotypic changes were observed. Spore spots were allowed to dry and then ~ 500 phages of interest were spotted onto one edge of each spore spot. Plates were allowed to dry and placed face-down on the glass of an Epson Canada Perfection V850 Pro Scanner—B11B224201 and pictures were taken as described above, with a focus on the timepoints of interest found using the large-scale method above. Pictures were taken of each strain every 12 h for 36 h total. Each phage was plated on the strains it was best able to replicate in and for which there was a noted phenotype. All phages were plated on *S. coelicolor* and WAC170; phage ϕScoe25 was plated on WAC178; and phages ϕScoe23, ϕScoe54, ϕScoe15, ϕScoe25, and ϕScoe55 were plated on WAC288. Each strain was also plated in the absence of phage. *S. coelicolor* was plated on ISP-2 tap medium; WAC288 and WAC170 were plated on ISP-2 medium; and WAC178 was plated on MYM MiliQ medium.

To determine the identity of the colored compounds produced in *S. coelicolor* upon phage infection, wild-type, Δ*actI*, and Δ *redD S. coelicolor* spores were plated on ISP-2 tap medium and challenged with phages ϕScoe15 and ϕScoe44 as described above. Experiments were performed in triplicate.

## Supplementary Information

Supplementary Information.

## Data Availability

All data generated or analysed during this study are included in this published article and its Supplementary Information files. Phage genomes are available in GenBank with accession numbers as shown in Table 1.
