# Supplementary material for: Complete genomes and comparative analyses of Streptomyces phages that influence secondary metabolism and sporulation
Source: Sci Rep. 2023 Jun 17;13:9820. doi: 10.1038/s41598-023-36938-z (PMC10276819; doi:10.1038/s41598-023-36938-z)
Supplement: Supplementary file 1 — Supplementary Information. [file 41598_2023_36938_MOESM1_ESM.pdf]

Supplementary Figure S1. Similarity of phage genomes

a

	ϕScoe25	ϕScoe15	ϕScoe3	ϕJoe	ϕScoe56	ϕScoe2	ϕScoe45	ϕScoe54	ϕScoe10	ϕScoe55	ϕScoe44	ϕScoe23	ϕScoe1	R4
**ϕScoe25**	100	91	83	86	84	86	85	86	80	82	81	75	74	72
**ϕScoe15**	91	100	83	85	84	86	84	86	80	81	81	76	74	73
**ϕScoe3**	83	83	100	86	87	84	87	85	81	84	84	75	73	73
**ϕJoe**	86	85	86	100	89	88	87	86	80	83	81	76	74	73
**ϕScoe56**	85	85	88	89	100	86	88	86	80	85	83	76	73	73
**ϕScoe2**	87	86	85	87	86	100	86	86	80	83	81	75	72	72
**ϕScoe45**	85	85	87	87	88	87	100	88	81	85	84	74	73	72
**ϕScoe54**	86	86	84	86	86	86	87	100	80	83	82	75	74	72
**ϕScoe10**	80	80	81	80	80	80	80	80	100	81	80	77	73	72
**ϕScoe55**	82	82	83	83	84	83	85	82	81	100	85	76	73	73
**ϕScoe44**	81	80	83	81	82	82	84	82	80	85	100	76	73	72
**ϕScoe23**	75	75	75	75	74	75	75	75	76	76	75	100	74	75
**ϕScoe1**	74	74	74	74	73	73	73	74	74	73	73	74	100	78
**R4**	72	73	72	72	72	72	72	72	71	71	71	74	78	100

b

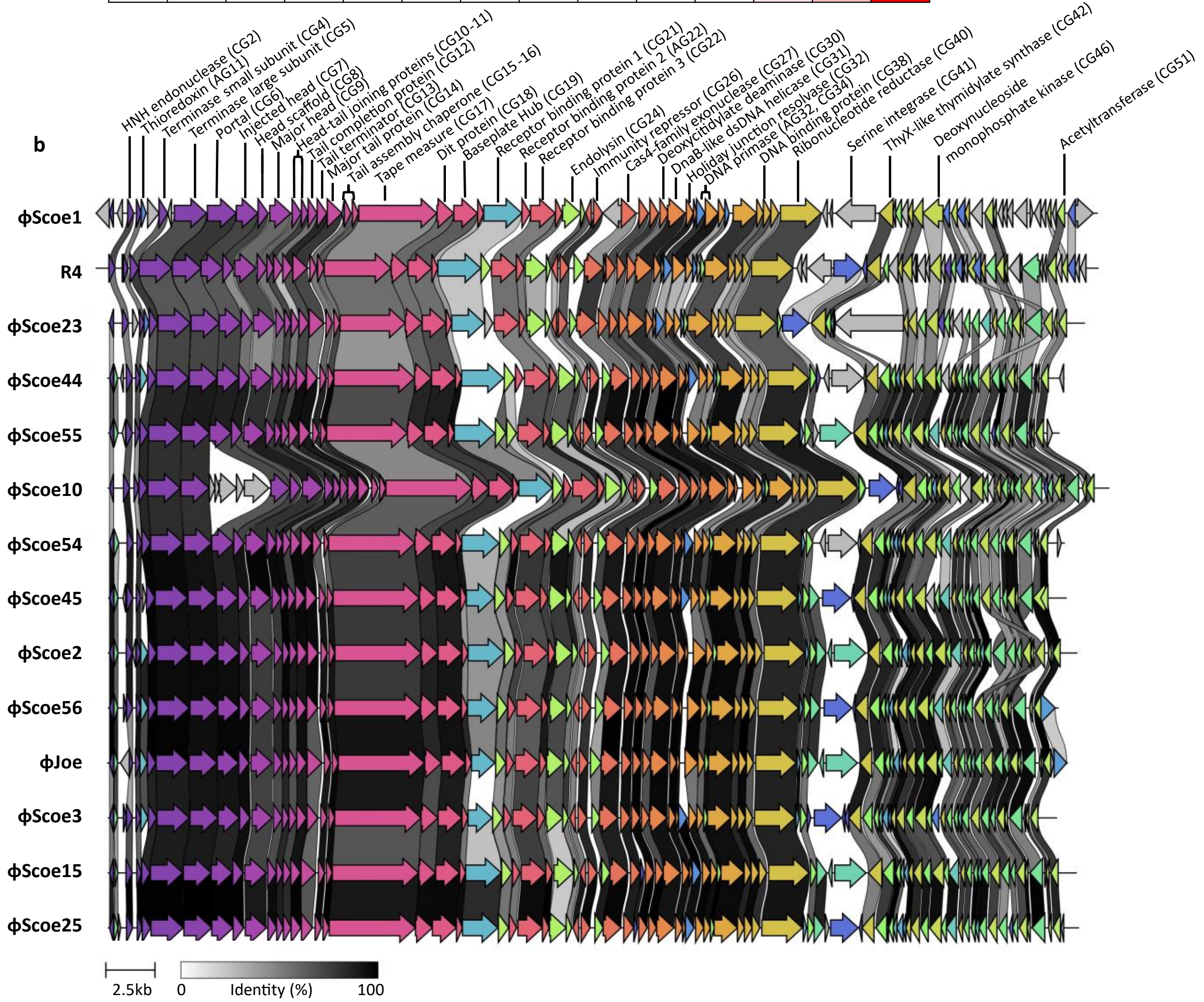

Fig. S1: Phage genomes are well-conserved. a) Average pairwise nucleotide identity matrix for 12 newly sequenced phage genomes and previously characterized phages ϕJoe and R4. Phages are organized by average percent identity. b) Alignment of phage genomes using Clinker (Gilchrist and Chooi 2021). Genes are colored by group based on similarity, and bars are shown between genes that share >30% identity and colored to reflect the percent identity. No bars are shown between the serine integrases and different colors are displayed due to their low pairwise percent identities. Genes between the HNH endonuclease and the endolysin are morphogenetic and largely conserved. All phages contain a serine integrase, endolysin, and receptor binding proteins, but many of these share <30% identity and are not connected. Phages are arranged by Clinker based on similarity; CG and AG refer to Supplementary Table S1 genes.

Supplementary Figure S2. Similarity of immunity repressors

a

	ϕScoe25	ϕScoe15	ϕScoe2	ϕJoe	ϕScoe3	ϕScoe54	ϕScoe56	ϕScoe55	ϕScoe45	ϕScoe44	ϕScoe10	ϕScoe1	R4	ϕScoe23
**ϕScoe25**	100	97	93	87	86	85	80	78	78	79	78	75	72	72
**ϕScoe15**	97	100	94	89	88	87	81	80	80	80	80	75	72	73
**ϕScoe2**	93	94	100	87	83	83	80	77	79	77	78	74	71	72
**ϕJoe**	87	89	87	100	90	90	84	85	86	85	78	72	70	70
**ϕScoe3**	86	88	83	90	100	89	86	87	87	86	77	71	68	72
**ϕScoe54**	85	87	83	90	89	100	88	89	89	88	77	72	70	70
**ϕScoe56**	80	81	80	84	86	88	100	87	87	89	75	69	64	72
**ϕScoe55**	78	80	77	85	87	89	87	100	97	93	77	70	67	70
**ϕScoe45**	78	80	79	86	87	89	87	97	100	93	78	70	67	70
**ϕScoe44**	79	80	77	85	86	88	89	93	93	100	76	69	65	70
**ϕScoe10**	78	80	78	78	77	77	75	77	78	76	100	79	73	78
**ϕScoe1**	75	75	74	72	71	72	69	70	70	69	79	100	83	75
**R4**	72	72	71	70	68	70	64	67	67	65	73	83	100	73
**ϕScoe23**	72	73	72	70	72	70	72	70	70	70	78	75	73	100

b

```
ϕScoe25 MGARKIVDEGEVIRWFEEGRTYAWMIEEYKRKYNIETVPSMWGNFRRRRGLQRRITRNDELIPWQVKAEHRWLYPIA
ϕScoe15 MGARKIVDEGEVIRWFEEGRTYAWMIEEYKRKYNIETVPSMWGNFRRRRGLQRRITRNDELIPWHVKPEHRWLYPIA
ϕScoe2  MGARKIVDEGEVIRWFEEGKTYAWMIEEYKRKYNIETVPSMWGNFRRRRGLQRRITRNDELIPWAVKPEHRWLYPIA
ϕJoe    MGARKIVDEGEVIRWFEEGRTYAWMIEEYRRKYNIETVPSMWGNFRRRRGLQRRITRNDELIPWAVKPEHRWLYPIG
ϕScoe3  MGARKIVDEGEVIRWFEEGRTYHWMVDEYRRKYNIETVPSLWGNFRRRRGLQRRITRNDELIPWQVKPEHRWLYPIG
ϕScoe54 MGARKIVDEGEVVRWFEEGRTYQWMIEEYRRKYNIETVPSMWGNFRRRRGLQRRITRNDELIPWQVKPEHRWLYPVG
ϕScoe56 MGARKIQDEGEVIRWFEEGKTYQWMVEEYRRKYNIETVPSLWGNFRRRRGLQRRITRNDELIPWQINPEHRWLYPVG
ϕScoe55 MGARKIQDEGEVLRWFEEGKTYQWMVEEYRRKYNIETVPSLWGNFRRRRGLDRRITRSDELIPWQVKPEHRWLYPVG
ϕScoe45 MGARKIQDEGEVMRWFEEGKTYQWMVEEYRRKYNIETVPSLWGNFRRRRGLDRRITRSDELIPWQVKPEHRWLYPVG
ϕScoe44 MGARKIQDEGEVMRWFEEGKTYQWMVEEYRRKYNIETVPSLWGNFRRRRGLDRRITRSDELIPWQVKPEHRWLYPVG
ϕScoe10 MGARKIQDEGEAIRWFEEGRTYHWMVEEYKRKYNIETVPSLWGNFRRRRGLDRRIVRNDDLIPWHVKPEHRWLYPVA
ϕScoe1  MGARKIQDEAEVIRWFEEGRTYQWMIDEYKRKYNIETVASMWGNFRRRRGLDRRIVRDDDLIPWFVKEEHRWAYPLA
R4      MGKRKIQDESEVVRWFEEGRTYAWMIAEYKRKYGIDTVPSMWGNFRRRRGLDRRIVRDDDLIPWFVKEEHRWAYPLA
ϕScoe23 MPARKIQNEQEVLRWFEEGRTYDWMVEEYKRKYNIETVPSLWGNFRRRRGLPRRIVRDDELIPWHIKEEHRWLYPLA

ϕScoe25 MLRAEARRREKGEEALTDLEKVRLPSWKKMLEDENAVVHYDPDTEEGFFYIPRQEGDDDLIHKPK-RVTKLRAADD--
ϕScoe15 MLRAEARRREKGEEALSDLEKVRLPSWKKMLEDENAVVHYDPDTEEGFFYIPRQEGDDDLIHKPK-RTTKLKAADD--
ϕScoe2  MLRAEARRREKGEEALSDLEKVRLPSWKTMLEEGAVVHYDPDTEEGFFYIPRQDGDDDLIHKPG-KTTKLRAAD---
ϕJoe    MLRVEARRREKGEEALSELEATRLASWKKMLTEENAVVHYDPETEEGFFYIPRQEGDDDLVHKPK-KSSRLKAAD---
ϕScoe3  MLRVEARRREKGEEALSELERTRLGSWLKMLADENAVVHYDPDTEEGFFYIPRGEGDDDLVHKPK-QTTRLKAAD---
ϕScoe54 MLRVEARRREKGEEALSELERTRLAAWKQMLLDQNAVVHYDPDTEEGFFYIPRQEGDTDFVHKPK-KTTRLKAAD---
ϕScoe56 MLRVEARRREKGEEALSELERTRLGAWKQMLQDNNAVVHYDPDTEEGFFYVPRQAGDKDLIHVPK-KSTRLKAVD---
ϕScoe55 MLRVEARRREKGESALSELEATRLAAWKQMLQDNNAVVHYDPETEAGFFYIPRGEGDTDLVHPPK-QTTRLKAAD---
ϕScoe45 MLRVEARRREKGEGALSELEATRLAAWKQMLQENNAVVHYDPETEEGFFYIPRGEGDTDLVHPPK-QTTRLKAAD---
ϕScoe44 MLRVEARRREKGEGALSELEATRLAAWKQMLADNNAVVHYDPDTEAGFFYIPRSEGDGDLVHKPK-VSTRLKAVD---
ϕScoe10 MLRIEARDRAG--APLNDDEKKRLASWKQMLTEQNAVVHYDPETEEGFFYVPRSEGDDDLIHKPKVKTTPRPAAD---
ϕScoe1  MLRAEARRRAG--KDITETDKARLASWHEMLKESNAVVHYDPDTEDGFFYVPRQEGDDDLIHRPKEKTTPRPNADKQE
R4      MLRAEARRRAG--KELTETDTSRLASWLDMLKDENAVVHYDPETDEGFFYIPRQPEDDDIIHRPDEKTTPRPNADRE-
ϕScoe23 MLRVEARKRAG--APVTELEESRLTNWRAMLEEENAVVHYDPDTEEGFFYVPREPGDDDIIHKPKRKTTPRPRRD---
```

Fig. S2: Pairwise percent identity matrix (a) and sequence alignment (b) for immunity repressor proteins among the 12 reported phages and closely related phages ϕJoe and R4. Darker red boxes in the matrix indicate higher percent identity. Shading of the matrix is normalized to maximum and minimum values present. Phages are arranged by similarity of the noted protein.

Supplementary Figure S3. Similarity of serine integrases and potential attP sites

a

	ϕScoe45	ϕScoe10	ϕScoe23	ϕScoe56	ϕScoe25	R4	ϕScoe3	ϕScoe54	ϕScoe44	ϕScoe1	ϕScoe15	ϕScoe2	ϕScoe55	ϕJoe
**ϕScoe45**	100	84	80	69	67	36	33	23	20	19	18	18	19	18
**ϕScoe10**	84	100	80	70	67	36	32	25	19	18	18	18	19	18
**ϕScoe23**	80	80	100	67	64	35	33	25	20	18	17	16	18	18
**ϕScoe56**	69	70	67	100	65	35	32	23	19	17	18	18	19	17
**ϕScoe25**	67	67	64	65	100	35	31	24	20	19	18	20	20	19
**R4**	36	36	35	35	35	100	42	26	19	15	19	18	21	21
**ϕScoe3**	33	32	33	32	31	42	100	25	16	17	19	19	18	19
**ϕScoe54**	23	25	25	23	24	26	25	100	15	17	17	17	16	16
**ϕScoe44**	20	19	20	19	20	19	16	15	100	14	17	17	16	16
**ϕScoe1**	19	18	18	17	19	15	17	17	14	100	16	17	14	14
**ϕScoe15**	18	18	17	18	18	19	19	17	17	16	100	91	76	78
**ϕScoe2**	18	18	16	18	20	18	19	17	17	17	91	100	75	78
**ϕScoe55**	19	19	18	19	20	21	18	16	16	14	76	75	100	85
**ϕJoe**	18	18	18	17	19	21	19	16	16	14	78	78	85	100

b

Phage	Sequence of attP site BLASTed	Percent Identity of Integrase with ϕjoe Integrase
**ϕJoe**	AGTTGTGGCCATGTGTCCATCTGGGGGCAGATGGAGACGGGGTCACATCC	100%
**R4**	GAAGCAGTGGTA	18%
**ϕBT1**	AGACGTTTCGGGTGCTGGGTTGTTGTCTCTGGACAGTGATCCATGGGAAACTACTCAGCACCACCAATGTTCC	14%
**ϕC31**	CCCCAACTGGGGTAACCTTTGAGTTCTCTCAGTTGGGG	11%
**TG1**	TCCAGCCCAACAGTGTTAGTCTTTGCTCTTACCCAGTTGGGCGGGA	13%
**SV1**	ATGTGGTCCTTTAGATCCACTGACGTGGGTCAGTGTCTCTAAAGGACTCGCG	14%

c

Phage	Sequence of hit	Location relative to integrase	Percent Identity with ϕJoe attP
**ϕScoe2**	AGTTGTGGCCATGTGTCCATCTGGGG **C**CAGATGGA**T**ACGGGGTCACA**A**CC	17 bp upstream	94%
**ϕScoe15**	AGTTGTGGCCATGTGTCCATCTGGGGGCAGATGG **CA**ACGGGGTCACA**A**CC	17 bp upstream	94%
**ϕScoe55**	AGTTGTGGCCATGTGTCCATCTGGGGGCAGATGGA **TG**C**A**GGGTCACA**A**CC	18 bp upstream	92%
**ϕJoe**	AGTTGTGGCCATGTGTCCATCTGGGGGCAGATGGAGACGGGGTCACATCC	18 bp upstream	N/A

Fig. S3: Similarity of intergenic DNA adjacent to the serine integrase of each phage to known and characterized attP sites from *Streptomyces* phages. a) Pairwise percent identity matrix for serine integrases among the 12 ϕ Scoe phages and closely related phages ϕJoe and R4. Darker red boxes in the matrix indicate higher percent identity. Shading of the matrix is normalized to maximum and minimum values present. Phages are arranged by similarity of the noted protein. b) Summary of attP sites BLASTed against phage genomes to find similarity. c) Detailed summary of hits from BLAST search of DNA from each phage against 6 characterized attP sites. All reported hits have >70% identity over the entire site. Standard BLAST parameters for slightly similar sequences were used.

Supplementary Figure S4. Infection of *S. coelicolor* lawn after incubation at 30 °C

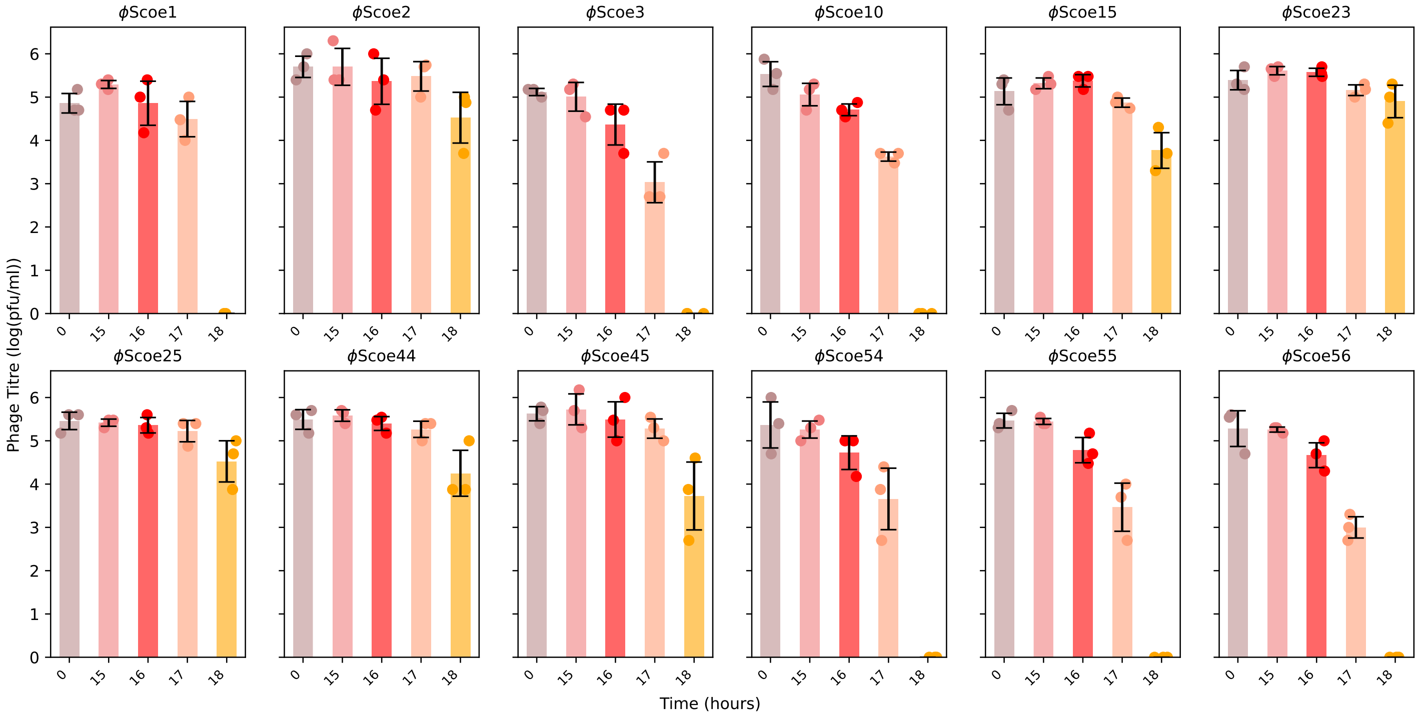

Fig. S4: Infection of S. coelicolor by each phage over time. 10-fold dilutions of each phage starting at ~$3x10^4$ pfu/mL were spotted on a lawn of $3x10^7$ S. coelicolor spores after 0, 15, 16, 17, or 18 hours of growth at 30 °C. Shown are graphs of the average log(titre) of each phage on S. coelicolor at each timepoint over 3 biological replicates. Error bars represent the standard deviation of the log(titre) over the replicates.

Supplementary Table S1. Genes present in each phage as shown in Fig. 2 and Fig. 3

	Phage Genomes												Putative Function or Sequence Similarity
	φScoe1	φScoe23	φScoe56	φScoe45	φScoe3	φScoe2	φScoe54	φScoe15	φScoe25	φScoe55	φScoe44	φScoe10	
AG1	AG1												
CG1													
AG2	AG2												
AG3			AG3	AG3	AG3	AG3	AG3	AG3	AG3	AG3	AG3		
AG4			AG4		AG4								
AG5				AG5									
AG6									AG6				
AG7										AG7			
AG8											AG8		
CG2													HNH endonuclease
AG9		AG9											
AG10						AG10							
CG3													
AG11	AG11	AG11											Thioredoxin
AG12	AG12												
AG13			AG13		AG13	AG13					AG13		Nuclease
AG14				AG14									
CG4													Terminase small subunit
AG15	AG15												
CG5													Terminase large subunit
CG6													Portal
AG16												AG16	
AG17												AG17	
AG18												AG18	
AG19												AG19	
AG20												AG20	
CG7													Injected head
CG8													Head scaffold
CG9													Major head
CG10													Head-tail joining protein 1
CG11													Head-tail joining protein 2
CG12													Tail completion protein
CG13													Tail terminator
CG14													Major tail
AG21	AG21												
CG15													Tail assembly chaperone
CG16													Tail assembly chaperone
CG17													Tape measure
CG18													Dit protein
CG19													Baseplate Hub
CG20													
CG21													Receptor binding protein 1
AG22	AG22		AG22	AG22	AG22	AG22	AG22	AG22	AG22		AG22	AG22	Receptor binding protein 2
AG23			AG23	AG23	AG23	AG23	AG23	AG23	AG23	AG23	AG23	AG23	
AG24										AG24			
AG25		AG25											
CG22													Receptor binding protein 3
CG23													
CG24													Endolysin
AG26	AG26												
AG27		AG27	AG27	AG27	AG27	AG27	AG27	AG27	AG27	AG27	AG27	AG27	
CG25													
CG26													Immunity repressor
AG28	AG28												
AG29		AG29	AG29	AG29	AG29	AG29	AG29	AG29	AG29	AG29	AG29	AG29	
AG30								AG30					
CG27													Cas4 family exonuclease
CG28													
AG31								AG31	AG31				
CG29													
CG30													Deoxycytidylate deaminase
CG31													DnaB-like dsDNA helicase
CG32													Holliday junction resolvase
CG33													

AG32	AG32	AG32		AG32	AG32		AG32	AG32	AG32		AG32		DNA primase
CG34													DNA primase
CG35													
AG33	AG33	AG33									AG33	AG33	
AG34		AG34	AG34	AG34	AG34	AG34	AG34	AG34	AG34	AG34	AG34	AG34	
CG36													
CG37													
CG38													DNA-binding protein
CG39													
CG40													Ribonucleotide reductase
AG35	AG35												
AG36	AG36												
AG37			AG37	AG37	AG37	AG37	AG37		AG37	AG37			
AG38		AG38	AG38	AG38	AG38	AG38	AG38	AG38	AG38	AG38	AG38	AG38	Lsr2-like DNA bridging protein
AG39			AG39	AG39		AG39		AG39	AG39				
AG40						AG40		AG40					
AG41					AG41						AG41		
AG42											AG42		
AG43							AG43						
AG44									AG44				
AG45										AG45			
CG41													Serine integrase
AG46			AG46	AG46	AG46				AG46			AG46	
AG47					AG47							AG47	
AG48							AG48						
AG49											AG49		
CG42													Thyx-like thymidylate synthase
AG50	AG50												
AG51			AG51	AG51	AG51	AG51	AG51	AG51	AG51	AG51	AG51	AG51	
AG52								AG52	AG52				
CG43													
AG53	AG53												
AG54		AG54	AG54	AG54	AG54	AG54	AG54	AG54	AG54				
AG55			AG55	AG55	AG55	AG55	AG55	AG55	AG55	AG55	AG55	AG55	
AG56					AG56	AG56	AG56		AG56				
AG57		AG57											
AG58		AG58											DNA polymerase III subunit alpha
CG44													
CG45													
AG59		AG59	AG59	AG59	AG59	AG59	AG59	AG59	AG59	AG59	AG59	AG59	
AG60												AG60	
CG46													Deoxynucleoside monophosphate kinase
AG61		AG61											
AG62				AG62									Endonuclease
AG63		AG63	AG63	AG63	AG63	AG63	AG63	AG63	AG63	AG63	AG63	AG63	
AG64	AG64				AG64								
CG47													
AG65	AG65												
AG66		AG66											
AG67		AG66	AG67	AG67		AG67	AG67	AG67	AG67	AG67			HNH endonuclease
AG68											AG68		
AG69												AG69	
AG70	AG70												
AG71		AG71											
AG72		AG72	AG72	AG72	AG72	AG72	AG72	AG72	AG72	AG72	AG72	AG72	
AG73			AG73	AG73	AG73	AG73	AG73	AG73	AG73	AG73	AG73	AG73	
AG74			AG74	AG74	AG74	AG74		AG74	AG74	AG74	AG74		
AG75		AG75	AG75	AG75	AG75	AG75	AG75	AG75	AG75	AG75	AG75	AG75	
AG76		AG76	AG76	AG76	AG76	AG76	AG76	AG76	AG76	AG76	AG76	AG76	
AG77		AG77	AG77	AG77	AG77	AG77	AG77	AG77	AG77	AG77	AG77	AG77	Ocr-like antirestriction protein
AG78									AG78	AG78		AG78	
AG79		AG79	AG79	AG79	AG79	AG79	AG79	AG79	AG79	AG79	AG79	AG79	
CG48													
CG49													
AG80	AG80												
AG81	AG81												
AG82	AG82												

AG83	AG83												
AG84	AG84												
AG85	AG85												
AG86	AG86												
AG87					AG87		AG87			AG87	AG87		
AG88								AG88					
CG50													
AG89	AG89												
CG51													Acetyltransferase
AG90	AG90												
AG91	AG91												
AG92			AG92										
AG93							AG93						
AG94									AG94				
AG95										AG95			
AG96											AG96		

Supplementary Table S2. Average pairwise percent identity of conserved morphogenetic genes

**Gene Product**	**Average Pairwise % Identity**
Terminase small subunit (CG4)	85.77%
Terminase large subunit (CG5)	87.24%
Portal (CG6)	86.14%
Injected head (CG7)	80.61%
Head scaffold (CG8)	79.75%
Major head (CG9)	75.82%
Head-tail joining protein 1 (CG10)	77.21%
Head-tail joining protein 2 (CG11)	84.87%
Tail completion protein (CG12)	64.89%
Tail terminator (CG13)	86.15%
Major tail (CG14)	80.21%
Tail assembly chaperone (CG15)	80.96%
Tail assembly chaperone (CG16)	82.70%
Tape measure (CG17)	62.21%
Dit protein (CG18)	76.98%
Baseplate Hub (CG19)	81.81%
Receptor binding protein (CG21)	34.13%
Receptor binding protein (AG22)	67.76%
Receptor binding protein (CG22)	69.70%

Supplementary Table S3. Summary of known stoperators in ϕScoe genomes

ΦScoe_phage	stoperator_source	intergenic_hits	known_motif
ΦScoe1	ELB20	19	TGTGCAAGTGTNGCA
ΦScoe1	L5	2	GGTGGCTGTCAAG
ΦScoe1	R4	20	TGTGCAAGTGTNGC
ΦScoe1	Zemlya	5	GTGTGCAACCTTCGTA
ΦScoe1	ΦHau3	2	AGTGTGTTAGNCNGAC
ΦScoe10	ELB20	9	TGTGCAAGTGTNGCA
ΦScoe10	L5	2	GGTGGCTGTCAAG
ΦScoe10	R4	11	TGTGCAAGTGTNGC
ΦScoe10	Zemlya	8	GTGTGCAACCTTCGTA
ΦScoe10	ΦHau3	9	AGTGTGTTAGNCNGAC
ΦScoe15	ELB20	6	TGTGCAAGTGTNGCA
ΦScoe15	L5	2	GGTGGCTGTCAAG
ΦScoe15	R4	10	TGTGCAAGTGTNGC
ΦScoe15	Zemlya	3	GTGTGCAACCTTCGTA
ΦScoe15	ΦHau3	4	AGTGTGTTAGNCNGAC
ΦScoe2	ELB20	5	TGTGCAAGTGTNGCA
ΦScoe2	L5	2	GGTGGCTGTCAAG
ΦScoe2	R4	9	TGTGCAAGTGTNGC
ΦScoe2	Zemlya	3	GTGTGCAACCTTCGTA
ΦScoe2	ΦHau3	4	AGTGTGTTAGNCNGAC
ΦScoe23	ELB20	11	TGTGCAAGTGTNGCA
ΦScoe23	L5	4	GGTGGCTGTCAAG
ΦScoe23	R4	12	TGTGCAAGTGTNGC
ΦScoe23	Zemlya	15	GTGTGCAACCTTCGTA
ΦScoe23	ΦHau3	6	AGTGTGTTAGNCNGAC
ΦScoe25	ELB20	3	TGTGCAAGTGTNGCA
ΦScoe25	L5	0	GGTGGCTGTCAAG
ΦScoe25	R4	8	TGTGCAAGTGTNGC
ΦScoe25	Zemlya	2	GTGTGCAACCTTCGTA
ΦScoe25	ΦHau3	5	AGTGTGTTAGNCNGAC
ΦScoe3	ELB20	4	TGTGCAAGTGTNGCA
ΦScoe3	L5	4	GGTGGCTGTCAAG
ΦScoe3	R4	8	TGTGCAAGTGTNGC
ΦScoe3	Zemlya	2	GTGTGCAACCTTCGTA
ΦScoe3	ΦHau3	5	AGTGTGTTAGNCNGAC
ΦScoe44	ELB20	5	TGTGCAAGTGTNGCA
ΦScoe44	L5	4	GGTGGCTGTCAAG
ΦScoe44	R4	8	TGTGCAAGTGTNGC
ΦScoe44	Zemlya	2	GTGTGCAACCTTCGTA
ΦScoe44	ΦHau3	4	AGTGTGTTAGNCNGAC
ΦScoe45	ELB20	5	TGTGCAAGTGTNGCA
ΦScoe45	L5	3	GGTGGCTGTCAAG
ΦScoe45	R4	9	TGTGCAAGTGTNGC

ΦScoe45	Zemlya	1	GTGTGCAACCTTCGTA
ΦScoe45	ΦHau3	5	AGTGTGTTAGNCNGAC
ΦScoe54	ELB20	5	TGTGCAAGTGTNGCA
ΦScoe54	L5	7	GGTGGCTGTCAAG
ΦScoe54	R4	9	TGTGCAAGTGTNGC
ΦScoe54	Zemlya	1	GTGTGCAACCTTCGTA
ΦScoe54	ΦHau3	3	AGTGTGTTAGNCNGAC
ΦScoe55	ELB20	3	TGTGCAAGTGTNGCA
ΦScoe55	L5	3	GGTGGCTGTCAAG
ΦScoe55	R4	5	TGTGCAAGTGTNGC
ΦScoe55	Zemlya	0	GTGTGCAACCTTCGTA
ΦScoe55	ΦHau3	3	AGTGTGTTAGNCNGAC
ΦScoe56	ELB20	2	TGTGCAAGTGTNGCA
ΦScoe56	L5	2	GGTGGCTGTCAAG
ΦScoe56	R4	5	TGTGCAAGTGTNGC
ΦScoe56	Zemlya	3	GTGTGCAACCTTCGTA
ΦScoe56	ΦHau3	5	AGTGTGTTAGNCNGAC
